# Associations between estimation of salt intake and salt-restriction spoons and hypertension status in patients with poorly controlled hypertension: a community-based study from Huzhou City, Eastern China

**DOI:** 10.1186/s12937-024-00912-w

**Published:** 2024-01-15

**Authors:** Qi Zhang, Yimei Shen, Meihua Yu, Zhongrong Yang, Zheng Huang, Jingying Ding, Xinfeng Zhu

**Affiliations:** Huzhou Center for Disease Control and Prevention, Huzhou, Zhejiang Province China

**Keywords:** Poorly controlled hypertension, Estimated salt intake, Spot urine, Salt-restriction spoon, Knowledge, Attitudes, Behaviors

## Abstract

**Background:**

As the prevalence of hypertension increases in China, it is advised to use salt-restriction spoons (SRS) as a lifestyle modification. This study aimed to examine the associations between estimated salt consumption, SRS usage, and the hypertension status in individuals with poorly controlled hypertension.

**Methods:**

Data was collected in Huzhou City, Zhejiang Province, in 2021 using convenience sampling. The analysis involved ordinal logistic regression and restricted cubic splines to assess the relevant factors.

**Results:**

The study found that 73.34% of the 1215 patients had uncontrolled blood pressure (BP). Urinary excretion was assessed through the utilization of the Kawasaki, INTERSALT, and Tanaka formulas. The outcomes of these three methodologies revealed average daily sodium excretion values of 208.70 (65.65), 154.78 (33.91), and 162.61 (40.87) mmol, respectively. The prevalence of utilizing SRS was found to be 37.78% in this study. Despite the acknowledgment among SRS users of the potential hazards associated with excessive salt consumption, there exists a contradictory pattern of attitudes and behaviors concerning salt reduction. Among individuals with different levels of salt intake (quartiles 1–4, Q1 vs Q4), there was a positive association between limiting salt and hypertension status when controlling for other variables (Kawasaki adjusted OR = 0.58, 95% CI = 0.43–0.79; INTERSALT adjusted OR = 0.62, 95% CI = 0.41–0.92; Tanaka adjusted OR = 0.61, 95% CI = 0.45–0.92, *p* < 0.05). Our research also revealed that using or used SRS was a protective factor for blood BP control (adjusted OR = 0.79, 95% CI = 0.64–0.99, *P* < 0.05). The restricted cubic spline plots illustrated a monotonic upward relationship between estimated 24-h urinary Na and BP (P-overall association < 0.05; P-non-linear association > 0.05).

**Conclusions:**

The use of dietary SRS could result in decrease in daily salt intake for BP control in patients with poorly controlled hypertension. To reduce the impact of high BP in China, additional studies are required to create interventions that can enhance the results for patients.

## Introduction

The relationship between salt and blood pressure (BP) has been characterized as a nuanced equilibrium, and the precise nature of the dose–response association remains a subject of debate [1–3]. Extensive evidence indicates that a diet high in salt leads to increased BP, a prominent risk factor for cardiovascular disease (CVD) [4]. Global mortality data on CVD reveals that exceeding the recommended salt intake is accountable for approximately 1.65 million deaths each year [5]. Nevertheless, a minority of researchers have posited that the advantages of sodium restriction for individuals with normal BP are modest and may potentially raise blood lipid levels and mortality risk [6]. Notably, China's daily salt consumption surpasses 12 g [7], exceeding the recommended limit of 5 g per day set by the World Health Organization (WHO) [8]. In China, it is recommended to use salt restriction spoons (SRS) as part of interventions aimed at reducing salt consumption among hypertensive individuals [9]. SRS are available in various sizes and shapes (e.g., 2, 3, and 6 g), enabling users to regulate their salt intake by estimating the required amount [10]. Nevertheless, there is a dearth of epidemiological data concerning the correlation between salt consumption, the utilization of SRS, and the hypertensive status of patients with poorly controlled hypertension in China.

The most precise method for assessing salt intake is through the collection of urine over a 24-h period, despite the inconvenience and impracticality of this approach in population-based epidemiological surveys [11]. The burden placed on participants has led to the exploration of alternative, more manageable methods for estimating 24-h urinary sodium excretion using spot urine samples, such as the Kawasaki [12], INTERSALT [13], and Tanaka methods [14], which are commonly employed. Moreover, WHO advocates for the adoption of spot urine techniques as a viable approach to estimating salt consumption in developing countries [8]. As a result, our community-based epidemiological field study utilized three spot urine methods, one of which is the Tanaka method, which has been endorsed by the Japanese Society of Hypertension in their clinical practice guidelines, despite its deviation from accurate salt intake measurement [15]. The primary objectives of this research endeavor were to assess salt intake levels and explore potential correlations between sodium consumption and various factors, with a particular focus on hypertension status. We conducted additional analysis to examine the non-linear association between salt intake and BP. It should be noted that our restricted cubic spline regression model may not yield accurate estimates when spot urine methods are utilized. By utilizing this information, it is possible to estimate the salt intake of individuals with uncontrolled hypertension, thereby facilitating efforts to promote salt reduction in China.

## Materials and methods

### Participants

A cross-sectional study was carried out in twenty communities/villages spanning ten streets/towns. A total of 1215 patients aged 35–75 years, who had primary and poorly controlled hypertension based on two or more consecutive measurements, were recruited for follow-up from January to August 2021. These patients were referred by their primary care physician for the management of uncontrolled hypertension and had been taking antihypertensive medications for at least one year. To prevent the manifestation of specific conditions, individuals who had additional organic cardiopulmonary vascular diseases, secondary hypertension caused by systemic diseases, psychiatric disorders, or intellectual disability were excluded from the study. Participants were requested to fulfill a standardized questionnaire, undergo a physical examination, and undergo laboratory testing. The research protocol received approval from the Ethical Review Committee of the Huzhou Center for Disease Control and Prevention.

### Standardized questionnaire

Trained data collectors administered a questionnaire to patients, gathering information on their (1) socio-demographic characteristics, (2) smoking and alcohol habits, physical activity levels, (3) current usage of antihypertensive medications, and (4) knowledge, attitude, and behavior regarding salt consumption and eating habits. Some of the questions were selected based on prior research [16]. Individuals who had smoked more than 100 cigarettes in the past were considered former smokers, while current smokers were those who had smoked at least one cigarette daily for six consecutive months. Alcohol consumption was determined by a response indicating a frequency of at least once per week in the previous year, while individuals who did not meet this criterion were considered nondrinkers. Physical activity was defined as ≥ 150 min per week of moderate-intensity or a combination of moderate and high-intensity exercise or ≥ 75 min of high-intensity exercise [16].

### Physical measurements

Following standardized protocols, measurements of height, weight, and BP were conducted. To ensure consistency in the measurement of height and weight for every participant, the Huachao Hi-Tech comprehensive height scale, with a precision of 0.1 cm for height and 0.1 kg for weight, was employed. BP was measured using the Omron HBP-1300 electronic sphygmomanometer with an accuracy of 1 mmHg, using the BP measurement method recommended in The Chinese Guidelines for the Prevention and Treatment of Hypertension (2018 edition) [9]. Patients are requested to seat for at least 5 min in a quiet room before BP measurements, and keep the upper arm at the heart level. Following the computation of the body mass index (BMI), the resulting values were categorized into various classifications based on Chinese guidelines, including underweight (BMI < 18.5), normal weight (18.5 ≤ BMI < 24), overweight (24 ≤ BMI < 28), and obese (BMI ≥ 28) [17]. Additionally, hypertension was classified into three distinct groups based on the recorded BP levels during this period, adhering to the global guidelines established by the International Society of Hypertension in 2020 [18]. The categories encompass the normal blood pressure group (Normal BP), which is defined by a BP reading below 140/90 mmHg. Additionally, there is the grade 1 hypertension group, characterized by a systolic blood pressure (SBP) ranging from 140 to 159 mmHg and/or a diastolic blood pressure (DBP) ranging from 90 to 99 mmHg. Lastly, there is the grade 2 hypertension group, characterized by a SBP equal to or exceeding 160 mmHg and a DBP equal to or exceeding 100 mmHg.

### Collection of urine and laboratory analysis

Respondents were given a 10 ml of standard urine collection container, and the fasting spot urine samples were obtained in the early morning after the first void, and all participants gave their informed consent by signing the necessary document. The urine samples were then deposited in 4 °C cooler containers and sent within 24 h to the central laboratory, where immediate analyses were conducted. C501 automatic biochemical analyzer (Roche Company of the United States) was utilized to detect urinary creatinine concentration by enzymatic method.

### Estimation of 24-h sodium excretion from spot urine samples

The Kawasaki formula [12] is as follows:

1 $${{\text{Pr}}}_{24{\text{hNa}}} =16.3\times \sqrt{{{\text{Su}}}_{{\text{Na}}}/{{\text{Su}}}_{{\text{Cr}}}\times {{\text{Pr}}}_{24{\text{hCr}}}}$$

Where Pr_24hCr_ for men = 15.12 × W + 7.39 × H – 12.63 × Y – 79.9 and for women = 8.58 × W + 5.09 × H – 4.72 × Y – 74.95.

The INTERSALT formula [13] is given as:

$${\text{Pr}}_{4\text{hNa}}\text{ }\mathrm{for}\;\mathrm{men}=\left(25.46+0.46\times{\text{Su}}_\text{Na}\right)-2.75\times{\text{Su}}_\text{Cr}-0.13\times{\text{Su}}_\text{k}+4.10\times\mathrm{BMI}+0.26\times{\text{Y}}_1,\;\mathrm{and}\;\mathrm{for}\;\mathrm{women}=\left(5.07+0.34\times{\text{Su}}_\text{Na}\right)-2.16\times{\text{Su}}_\text{Cr}-0.09\times{\text{Su}}_\text{k}+2.39\times\mathrm{BMI}+2.35\times{\text{Y}}_2-0.03\times{\text{Y}}_2$$

The Tanaka formula [14] is given as:

2 $${Pr}_{24hNa} =21.98 \times {\left({Su}_{Na}/{Su}_{Cr} \times {Pr}_{24hCr}\right)}^{0.392}$$

Where Pr_24hCr_ = 14.89 × W + 16.14 × H – 2.04 × Y – 2244.45.

In the above formula: Pr_24hNa_ is predicted 24-h urinary sodium excretion value (mmol/day); Pr_24hCr_ is predicted 24-h urinary creatinine excretion value (mg/day); Su_Na_ is spot urine sodium (mmol/l); Su_k_ is spot urine potassium (mg/day); Su_Cr_ is Spot urine concentration (mg/dl); W is weight (kg); H is high (cm); Y is age (years); BMI is body mass index (kg/m^2^).

By using the following equation, we can convert urine sodium excretion values (mmol/day) into urine salt excretion values (g/day) [19]:

3 $$\mathrm{Estimated}\;24h\;\mathrm{salt}\;\mathrm{intake}\;(\text{g}/\text{day})={Pr}_{24hNa}\times0.0585$$

### Statistical analysis

Statistical analyses were performed using SPSS software version 21.0 (IBM, Armonk, New York, United States), GraphPad Prism 8 software and R version 4.2.3. Normally distributed data were presented as the mean (standard deviation) through one-factor analysis of variance (ANOVA). Spot urine sodium, potassium, and concentration were reported as the median (M) and interquartile range (IQR) for non-normally distributed data, analyzed using the Kruskal–Wallis test. The chi-square test was employed to compare categorical variables among participants. A univariate ordinal logistic regression model was employed to examine the risk factors associated with hypertension among the participants. Following the univariate analysis, variables with a significance level of *p* < 0.1, along with sex, were incorporated as independent variables in the model, while hypertension status served as the dependent variable. The model successfully passed the parallelism test, and for the subsequent multivariate analysis, ordinal logistic regression was utilized. To assess the shape of the relationship between sodium and BP, we created restricted cubic spline plots adjusted for age, sex, region, BMI, alcohol consumption, and number of antihypertensive medications. Data were fitted by a linear regression model, and the model was conducted with 4 knots at the 5th, 35th, 65th, 95th percentiles of age (reference is the 5th percentile). Statistical significance was determined at a threshold of *p* < 0.05.

## Results

### General demographic characteristics of participants

Table 1 presents the descriptive analysis of the principal characteristics and laboratory parameters of the study population. 1215 patients with poorly controlled hypertension were eligible for inclusion in the study, of which 53.66% were men; 54.24% had a low education level (primary or below education), 60.33% were rural dwellers, and the mean (SD) age of the participants was 60.83 (7.76) years, the mean (SD) BMI was 25.96 (3.75) kg/m^2^. Regarding the hypertension status, normal BP, grade 1, and grade 2, the rates were 26.67%, 47.33%, and 26.01%, respectively. Of all participants, 37.78% had previously or were currently utilizing salt-restriction utensils, 37.86% had physical activity, 34.65% consumed alcohol, 62.72% were non-smokers. Participants who had taken multiple antihypertensive medications were more prone to have uncontrolled hypertension (*p* < 0.001). Mean SBP and DBP were 146.67 (16.33) mm Hg and 87.77 (9.85) mm Hg, respectively. The median (IQR) concentrations of sodium, potassium, and urine were 124.00 (70.00) mmol/L, 26.78 (20.82) mmol/L, and 10.01 (8.07) mg/dl, respectively.

**Table 1:** Socio-demographic and laboratory parameters characteristics of 1215 participants by classification of hypertension status

Variable	Normal BP (*n* = 324)	Grade 1 Hypertension (*n* = 575)	Grade 2 Hypertension (*n* = 316)	*p* Value
Age (years) (mean ± SD)	61.67 ± 7.57	60.41 ± 7.80	60.71 ± 7.82	0.060
Gender, n (%)				
Male	160 (49.38)	318 (55.30)	174 (55.06)	0.196
Female	164 (50.42)	257 (44.70)	142 (44.94)	
Region, n (%)				
Rural	165 (50.93)	219 (38.09)	98 (31.01)	0.000*
Urban	159 (49.07)	356 (61.09)	218 (68.99)	
Education Level				
Primary or below	174 (53.70)	320 (55.65)	165 (52.22)	0.513
Secondary	113 (34.88)	198 (34.43)	113 (35.76)	
Tertiary or above	37 (11.42)	57 (9.91)	38 (12.03)	
BMI groups (kg/m^2^), n (%)				
Underweight/Normal weight	117 (36.11)	167 (29.04)	76 (24.05)	0.004*
Overweight	145 (44.75)	270 (46.96)	146 (46.20)	
Obese	62 (19.14)	138 (24.00)	94 (29.75)	
SRS using or used, n (%)	131 (40.43)	229 (39.83)	99 (31.33)	0.023*
Physical activity, n (%)	128 (39.51)	219 (38.09)	113 (35.76)	0.613
Alcohol consumption, n (%)	83 (25.62)	211 (36.70)	127 (40.19)	0.000*
Smoking status, n (%)				
Never smoked	210 (64.81)	354 (61.57)	198 (62.66)	0.675
Former smoker	35 (10.80)	78 (13.57)	45 (14.24)	
Current smoker	79 (24.38)	143 (24.87)	73 (23.10)	
Number of antihypertensive drugs taken, n (%)				
1	241 (74.38)	394 (68.52)	190 (60.13)	0.000*
2	74 (22.84)	161 (28.00)	101 (31.96)	
≥ 3	9 (2.78)	20 (3.48)	25 (7.91)	
Systolic blood pressure, (mm Hg) (mean ± SD)	128.64 ± 8.31	146.28 ± 7.69	165.88 ± 12.03	0.000*
Diastolic blood pressure, (mm Hg) (mean ± SD)	79.3 1 ± 6.86	87.76 ± 7.22	96.47 ± 9.01	0.000*
Laboratory results (M(IQR))				
Su_Na_ (mmol/L)	116.00 (69.00)	123.00 (73.00)	134.00 (69.00)	0.002*
Su_k_ (mmol/L)	26.75 (23.43)	27.84 (22.24)	25.25 (17.14)	0.049*
Su_Cr_ (mg/dL)	9.57 (7.64)	10.61 (5.47)	9.54 (8.57)	0.102

Age, systolic blood pressure, and diastolic blood pressure are expressed as mean (SD), using one-factor analysis of variance (ANOVA); Su_Na_, Su_k_, Su_k_, and Su_Cr_ are expressed as the median (M) and interquartile range (IQR), which is used for data with non-normal distributions using Kruskal–Wallis test to compare

^*^p < 0.05

### Comparison of estimated 24-h urinary sodium excretion and salt intake

**Fig. 1:**
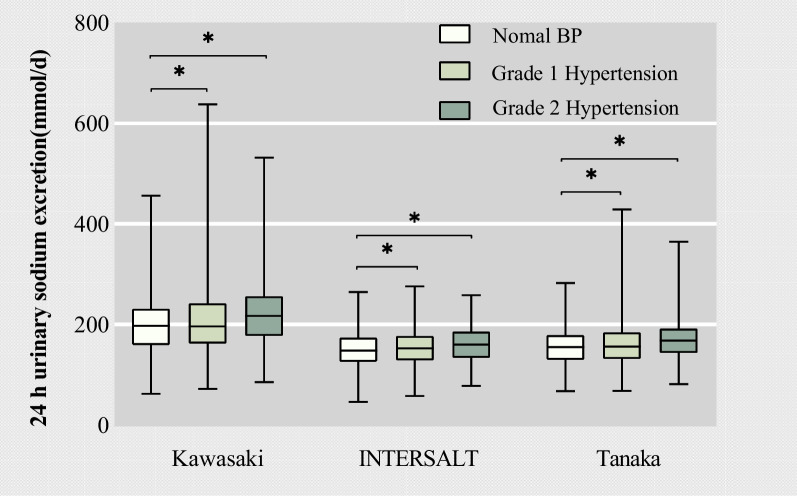
Distributions of the estimated 24-h urinary sodium excretion values by three methods according to hypertension status

The estimated 24-h sodium excretion mean (SD) values by the three methods (The Kawasaki, INTERSALT, and Tanaka formula) were 208.70 (65.65), 154.78 (33.91), and 162.61 (40.87) mmol/day, equal to a salt intake of 12.21 (3.85), 9.05 (1.99), and 9.51 (2.39) g/day, respectively. Figure 1 depicts the estimated distribution of 24-h urinary sodium excretion based on hypertension status, those who at grade 2 hypertension had the highest 24-h urinary sodium excretion of all hypertension grade groups (*p* < 0.001).

The average sodium consumption among males tended to be higher than that among females (INTERSALT method, 10.08 g/day vs 7.87 g/day, *p* < 0.001; Tanaka method, 9.68 g/day vs 9.36 g/day, *p* < 0.05; no difference in Kawasaki method, respectively). Individuals with higher levels of education exhibited lower sodium intake compared to those with primary education (Kawasaki method, 11.14 g/d vs 12.43 g/d, *p* < 0.001; INTERSALT method, 8.90 g/d vs 9.34 g/d, *p* < 0.001; Tanaka method, 8.73 g/d vs 9.71 g/d, *p* < 0.001, respectively). Obese participants had a higher daily sodium intake compared to underweight or normal-weight participants (Kawasaki method, 13.05 g/d vs 11.58 g/d, *p* < 0.001; INTERSALT method, 10.22 g/d vs 8.17 g/d, *p* < 0.001; Tanaka method, 10.07 g/d vs 9.10 g/d, *p* < 0.001, respectively), and individuals with grade 2 hypertension consumed more sodium than those with normal blood pressure (Kawasaki method, 12.97 g/day vs 11.69 g/day, *p* < 0.001; INTERSALT method, 9.40 g/day vs 8.74 g/day, *p* < 0.001; Tanaka method, 9.97 g/day vs 9.21 g/day, *p* < 0.001, respectively). Furthermore, participants who had previously used or were currently using SRS demonstrated a lower average daily sodium intake than those who had not (Kawasaki method, 12.03 g/day vs 12.94 g/day, *p* < 0.05; INTERSALT method, 8.86 g/day vs 9.42 g/day, *p* < 0.001, Tanaka method, 9.42 g/d vs 9.89 g/d, *p* < 0.05, respectively). Overall, responses exhibited a similar salt intake pattern, stratified by three methods (Table 2).

**Table 2:** Factors associated with salt intake by three methods in the study population

Variable	Kawasaki of Salt intake (g/day)	*p* Value	INTERSALT of Salt intake (g/day)	*p* Value	Tanaka of Salt intake (g/day)	*p* Value
Sex						
Male	12.41 ± 3.86	0.053	10.08 ± 1.79	0.000*	9.68 ± 2.48	0.020*
Female	11.99 ± 3.83		7.87 ± 1.48		9.36 ± 2.30	
Region						
Rural	12.34 ± 3.86	0.727	9.14 ± 1.86	0.002*	9.58 ± 2.37	0.711
Urban	12.02 ± 3.84		8.92 ± 2.16		9.41 ± 2.42	
Age groups						
35–44	12.83 ± 4.56	0.052	9.58 ± 1.72	0.000*	9.64 ± 2.71	0.158
45–59	11.87 ± 3.69		9.00 ± 1.87		9.28 ± 2.31	
60–75	12.32 ± 4.37		9.13 ± 1.94		9.53 ± 2.69	
Education Level						
Primary	12.43 ± 3.91	0.002*	9.34 ± 1.94	0.001*	9.71 ± 2.43	0.000*
Secondary	12.22 ± 3.89		8.93 ± 1.71		9.44 ± 2.39	
Tertiary or above	11.14 ± 3.22		8.90 ± 2.05		8.73 ± 2.01	
BMI groups (kg/m^2^)						
Underweight/Normal weight	11.58 ± 3.46	0.000*	8.17 ± 1.87	0.000*	9.10 ± 2.15	0.000*
Overweight	12.18 ± 3.99		9.01 ± 1.82		9.48 ± 2.47	
Obese	13.05 ± 3.89		10.22 ± 1.87		10.07 ± 2.43	
Hypertension status						
Normal BP	11.69 ± 3.48	0.000*	8.74 ± 1.93	0.000*	9.21 ± 2.19	0.000*
Grade 1 Hypertension	12.09 ± 3.96		9.04 ± 1.99		9.43 ± 2.48	
Grade 2 Hypertension	12.97 ± 3.91		9.40 ± 2.00		9.97 ± 2.36	
SRS Status						
Using or used	12.03 ± 3.57	0.0048*	8.86 ± 1.91	0.001*	9.42 ± 2.40	0.021*
Never use	12.94 ± 3.87		9.42 ± 2.03		9.89 ± 3.04	
Total	12.21 ± 3.85		9.05 ± 1.99		9.51 ± 2.39	

Data are presented as mean (SD). ANOVA was used to compare the salt intake of different characteristics of the participants

^*^p < 0.05

### Associations between estimation of salt intake and SRS and hypertension status in patients with poorly controlled hypertension

The results of the univariate analysis indicated that a lower salt intake (the Q1 vs Q4 of salt intake, Kawasaki method, crude OR = 0.52, *p* < 0.001; INTERSALT method, crude OR = 0.55, *P* < 0.001; Tanaka method, crude OR = 0.59, *P* < 0.001, respectively); an urban origin (crude OR = 0.56, *p* < 0.001); using or used SRS utensil (crude OR = 0.77, *p* < 0.05), were protective factors for BP control. It was also showed that body mass index (crude OR = 1.05, *p* < 0.05); alcohol consumption (crude OR = 1.55, *p* < 0.001); increasing number of medications (crude OR = 1.45, *p* < 0.001, 2 vs. 1; crude OR = 2.64, *p* < 0.002, 2 vs. ≥ 3) were risk factors for BP control (Table 3).

**Table 3:** Univariate analysis about the risk of hypertension among participants

Variable	Crude OR	95%CI	*p* Value
Male (vs. Female)	1.17	0.95–1.44	0.146
Urban (vs. Rural)	0.56	0.45–0.69	0.001*
Age (years)	0.99	0.98–1.01	0.090
BMI (kg/m2)	1.05	1.01–1.08	0.002*
SRS using or used (vs. Never use)	0.77	0.62–0.96	0.019*
Alcohol consumption (vs. Nondrinkers)	1.55	1.24–1.93	0.001*
Number of antihypertensive drugs taken, n (%)			
2 (vs. 1)	1.45	1.14–1.83	0.000*
≥ 3 (vs. 1)	2.64	1.57–4.46	0.002*
Salt intake Quartile			
Kawasaki method			
Q1(< 9.55) vs. Q4(> 14.24) (g/day)	0.52	0.39–0.71	0.001*
Q2(9.55–11.78) vs. Q4(> 14.24) (g/day)	0.61	0.45–0.82	0.001*
Q3(11.78–14.24) vs. Q4(> 14.24) (g/day)	0.67	0.50–0.91	0.009*
INTERSAL method			
Q1(< 7.54) vs. Q4(> 10.43) (g/day)	0.55	0.41–0.74	0.001*
Q2(7.54–8.91) vs. Q4(> 10.43) (g/day)	0.62	0.46–0.84	0.002*
Q3(8.91–10.43) vs. Q4(> 10.43) (g/day)	0.71	0.52–0.95	0.022*
Tanaka method			
Q1(< 7.80) vs. Q4(> 10.78) (g/day)	0.59	0.44–0.80	0.001*
Q2(7.80–9.27) (vs. Q4(> 10.78) (g/day)	0.60	0.44–0.81	0.001*
Q3(9.27 –10.78) vs. Q4(> 10.78) (g/day)	0.89	0.66–1.20	0.454

^*^p < 0.05

**Fig. 2:**
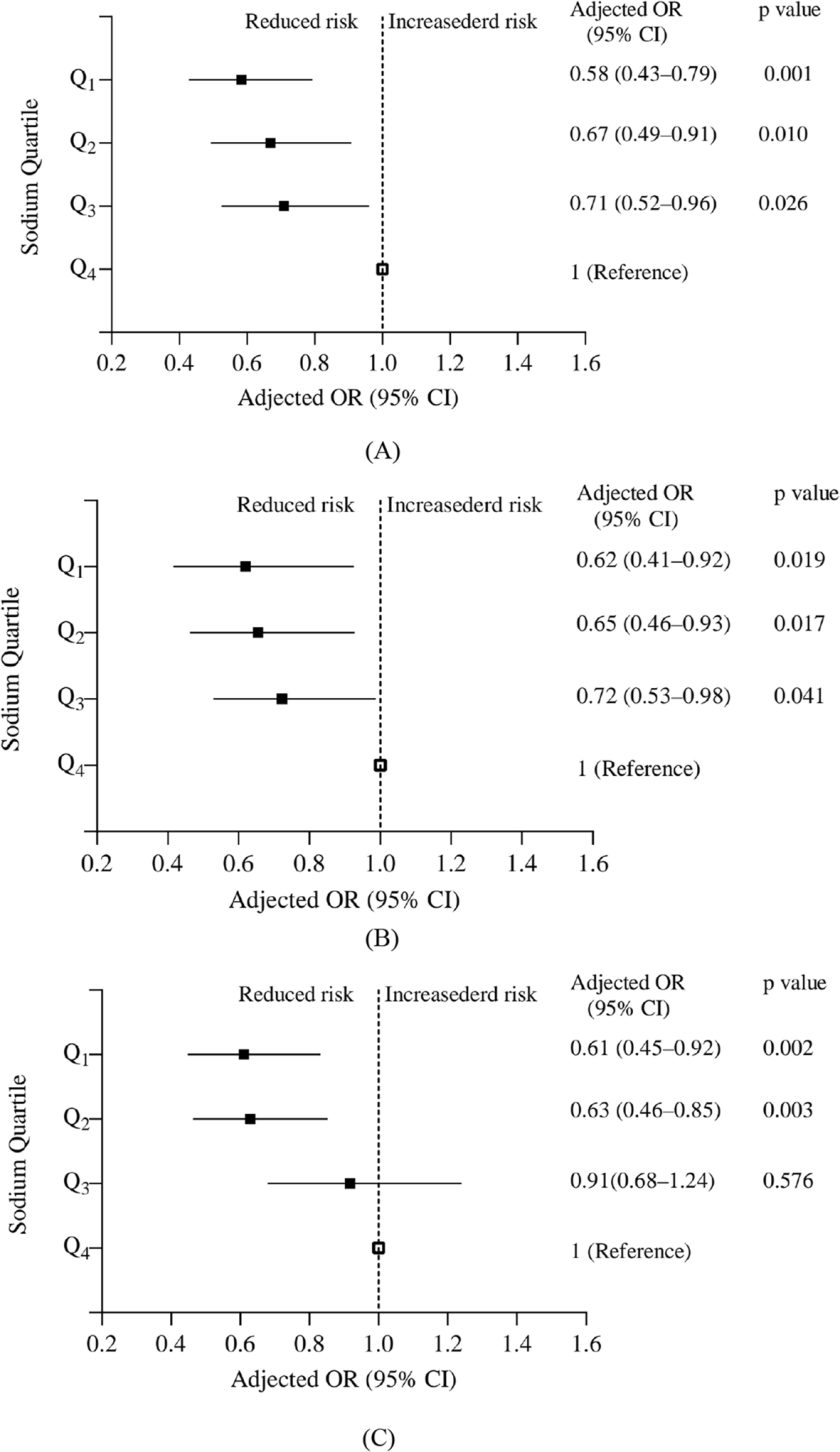
Associations between estimation of sodium (**A**) by Kawasaki method, (**B**) by INTERSAL method, (**C**) by Tanaka method, and hypertension status in patients with poorly controlled hypertension

After controlling for confounding variables such as age, sex, region, BMI, alcohol consumption, and number of antihypertensive medications, a multiple logistic regression analysis was employed to examine the statistical significance of differences in salt intake and SRS usage in relation to hypertension status. Figure 2 illustrates that individuals with a lower estimated salt intake (specifically, those in the first quartile compared to the fourth quartile) according to the three measurement methods were more inclined to exhibit a lower level of hypertension (Kawasaki adjusted OR = 0.58, 95% CI = 0.43–0.79, *P* < 0.001, INTERSALT adjusted OR = 0.62, 95% CI = 0.41–0.92, *p* < 0.05, Tanaka adjusted OR = 0.61, 95% CI = 0.45–0.92, *P* < 0.05). Our findings suggest that the utilization of a SRS for cooking was associated with a reduction in salt consumption, resulting in a deceleration of BP decline (adjusted OR = 0.79, 95% CI = 0.64–0.99, *P* < 0.05).

### Relationship between estimation of sodium and BP

**Fig. 3:**
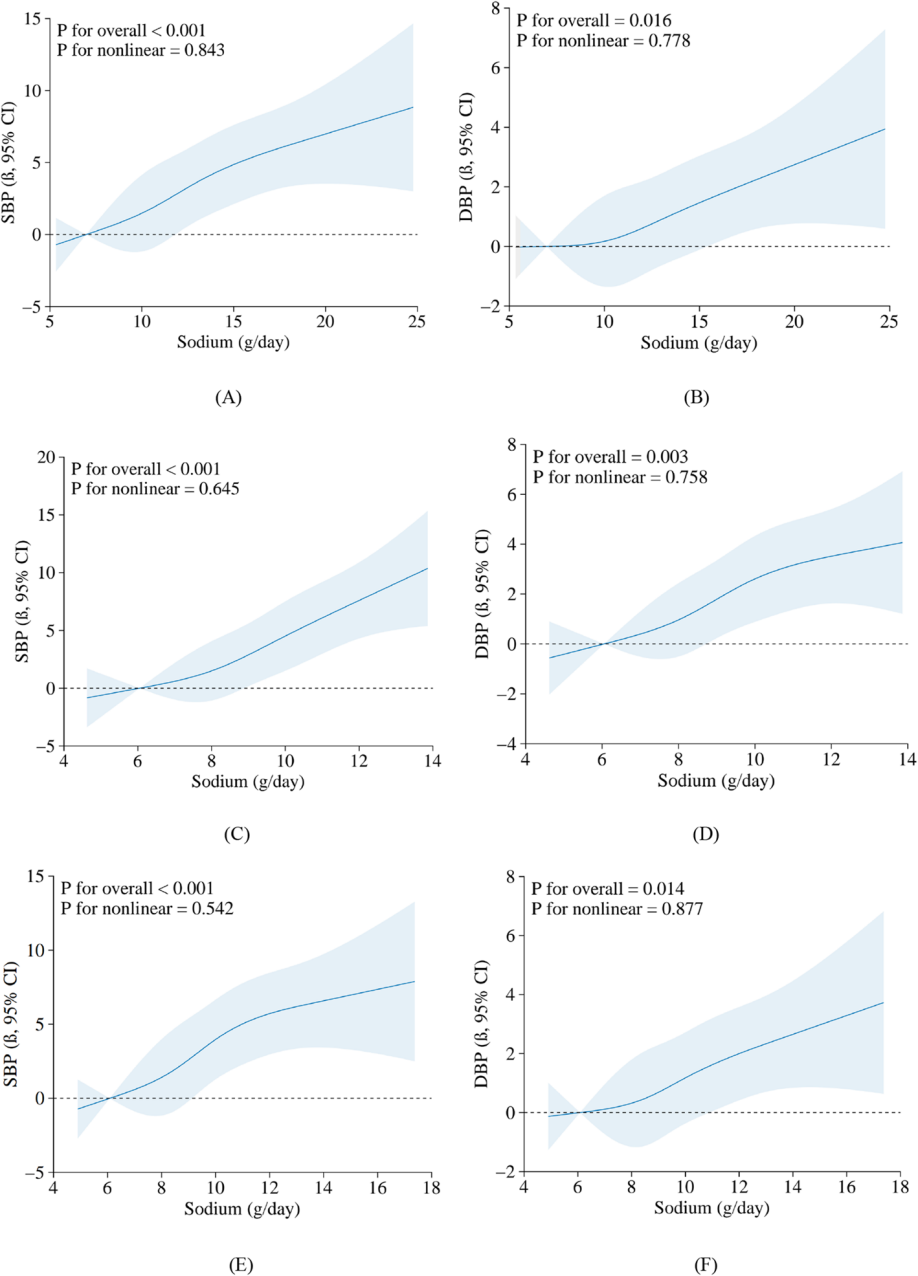
Association between estimation of sodium (**A**) and (**B**) by Kawasaki method, (**C**) and (**D**) by INTERSAL method, (**E**) and (**F**) by Tanaka method and SBP/DBP using a restricted cubic spline regression Model. Graphs show β for SBP or DBP according to sodium adjusted for age, sex, region, BMI, alcohol consumption, and number of antihypertensive medications. Data were fitted by a linear regression model, and the model was conducted with 4 knots at the 5th, 35th, 65th, 95th percentiles of age (reference is the 5th percentile). Solid lines indicate β, and shadow shape indicates 95% CI

Figure 3 illustrates the dose–responsive correlation between estimation of sodium and BP from restricted cubic splines with 4 knots at the 5th, 35th, 65th, 95th percentiles of sodium (reference is the 5th percentile). The β of SBP and DBP exhibited an upward trend as sodium levels increased (P-overall association < 0.05; P-non-linear association > 0.05). According to Kawasaki, INTERSAL, and Tanaka's formulas on urinary sodium measurement, the spline analysis showed comparable patterns in the correlation between sodium intake and BP, without any discernible flattening of the curve at extreme levels of sodium exposure. However, the magnitude of this association varied across different methods of urinary sodium measurement when employing the same statistical model.

### Questionnaire-based survey of knowledge, attitudes, and behaviors about salt intake by hypertension status

The findings indicate that a significant proportion of respondents (79.18%) demonstrated awareness regarding the adverse consequences associated with excessive salt consumption. Notably, individuals who had utilized SRS reported significantly higher levels of knowledge in this regard compared to those who had not used SRS (*p* < 0.001). It is worth mentioning that a considerable portion of researchers (37.78%) lacked awareness regarding the recommended daily sodium intake, which should ideally be less than 6 g of salt per day. Furthermore, a substantial majority of patients (88.48%) expressed agreement with the ongoing promotion of low-salt diets. With the exception of the misperception that low-sodium intake leads to decreased strength, which was approved by 33.50% of respondents, SRS users demonstrated significantly better performance in addressing this attitude question (*p* < 0.001). In terms of dietary behaviors, a significant majority of patients (78.35%) expressed an intention to decrease their salt intake, while a majority of participants (67.90%) reported regular consumption of salty condiments (more than 3–5 days per week). Consistently, SRS users exhibited significantly higher scores in knowledge, attitude, and behavior assessments compared to non-users (Table 4).

**Table 4:** Characteristics of knowledge, attitude, and behavior about salt intake by salt-restriction spoon status

Characteristic	Using or Used Salt-Restriction Spoons			
	All (*n* = 1215)	Yes (*n* = 459)	NO (n = 756)	*p* Value
Knowledge				
Know the recommended adult sodium consumption, n (%)	459 (37.78)	196 (42.70)	263 (34.79)	0.006*
High sodium consumption can cause or exacerbate obesity, hypertension, and cardiovascular disease, n (%)	962 (79.18)	396 (86.27)	566 (74.87)	0.000*
Attitude				
Consider that low-sodium diets cause weakness, n (%)	407 (33.50)	112 (24.40)	295 (39.02)	0.000*
A diet limited in sodium should be promoted, n (%)	1075 (88.48)	403 (87.80)	672 (88.89)	0.564
Behavior				
Consume condiments high in sodium frequently (monosodium glutamate, soy sauce), n (%)	825 (67.90)	261 (56.86)	564 (74.60)	0.000*
Make a concerted effort to cut down on salt use in the kitchen, n (%)	952 (78.35)	378 (82.35)	574 (75.93)	0.008*

The chi-square test was used to compare the characteristics of different SRS status. Percentages are column percent

^*^ *p* < 0.05

## Discussion

China is currently facing an escalating burden of hypertension, necessitating the implementation of lifestyle modifications and antihypertensive medications as the fundamental components of an effective hypertension management approach [20, 21]. Reducing salt consumption in the diet is of great significance among the different lifestyle measures targeting the reduction of noncommunicable diseases in society [4]. To achieve favorable outcomes, diverse state and community salt reduction strategies have been devised, such as the implementation of The National Essential Public Health Services Package, which was introduced by the Chinese government in 2009 [22]. This package offers a range of services, including health records management, screening, and follow-up, thereby contributing to the overall objective of salt reduction. In China, the primary origin of sodium consumption is salt utilized in household cooking, while in western nations, processed food plays a major role in contributing to dietary salt [23]. Consequently, there is a need for a public campaign aimed at reducing salt usage in cooking. Additionally, it is crucial to develop a suitable assessment methodology for measuring sodium intake and to provide a reference for the formulation of precise policies for the prevention of hypertension. This approach is essential for effectively controlling BP in individuals with hypertension.

Multiple studies have demonstrated that the spot urine method is a suitable approach for estimating 24-h urinary sodium excretion, enabling the investigation of the correlation between salt intake and hypertension and other diseases within the general population. Groenland E.H. et al. [24] employed Kawasaki formulae to assess the relationship between estimated salt intake and BP. The findings revealed that, for each 1 g/day augmentation in sodium urinary excretion, the average SBP and DBP increased by 1.28 mmHg (95% CI: 0.95–1.62) and 0.46 mmHg (95% CI: 0.28–0.65), respectively. In a study conducted by Goto A et al. [25], the risk of developing stomach cancer was assessed by comparing it to the estimated salt consumption obtained from spot urine using the Tanaka technique. Similarly, X F Du et al. [26] employed three spot urine methods to estimate 24-h urine sodium excretion among residents in Zhejiang Province and compared it to actual values (167.10 (74.70) mmol/day). The Kawasaki method yielded an overestimation of 184.61 (57.10) mmol/day, whereas the INTERSALT and Tanaka methods resulted in underestimations of 134.62 (39.21) and 143.20 (35.66) mmol/day, respectively.

According to our research, the sodium excretion values estimated using the Kawasaki, INTERSALT, and Tanaka formula for a 24-h period were 208.70 (65.65), 154.78 (33.91), and 162.61 (40.87) mmol/day, respectively. These values exceed the average sodium intake of Zhejiang Province residents [26]. Despite slight variations between the estimated sodium salt intake and the precise individual value, our study indicates that individuals with uncontrolled hypertension are part of the population with elevated sodium consumption. The habitual salt intake in China is approximately 12 g per day [7], and our study found that the average difference between higher (Kawasaki method is 12.21 g per day) and lower (9.05 g per day) salt intake was 3 g per day. Considering previous research, the Kawasaki method may overestimate the actual value of 24-h urinary sodium excretion, while the INTERSALT and Tanaka methods tend to underestimate it [26]. Despite these inherent inaccuracies, we believe that the findings of our study are applicable to real-life conditions. By reducing salt consumption by 7 g (equivalent to approximately one more large-sized SRS), individuals would approach the World Health Organization's recommended level of 5 g per day for the population [8]. Significantly, the estimations of salt consumption were notably elevated among individuals classified as obese, in comparison to those who were categorized as normal-weight or overweight. Likewise, upon stratifying the responses based on hypertension status, it was observed that participants diagnosed with Grade 2 hypertension exhibited the highest sodium intake. Previous studies have also documented the augmented sodium consumption observed in individuals with a higher body mass index, as well as inadequate BP regulation among hypertensive outpatients [19, 27].The potential mechanistic pathways through which insulin resistance and overweight may contribute to the development of isolated systolic hypertension involve an increase in salt sensitivity, leading to endothelial dysfunction, arterial rigidity, and elevated BP [28]. Furthermore, these pathways may be influenced by suboptimal dietary habits, including a preference for high-fat foods and the use of sodium as a flavor enhancer. Prior studies have shown that the addition of salty condiments to meals can enhance their taste, but this practice may carry the risk of excessive caloric intake and subsequent weight gain [29].

In order to mitigate salt intake across the entire population of China, the implementation of a multi-faceted initiative, including the adoption of SRS, has been widely employed [30]. In the present study, the prevalence of SRS utilization was determined to be 30.21%, surpassing the 12.0% reported in a previous survey conducted in 2017 among 7512 individuals residing in China's Zhejiang Province (of whom 35.3% were identified as hypertensive) [16]. Furthermore, the findings indicate that the use of SRS and hypertension status were positively associated with reduced sodium intake, rather than a lower sodium-to-potassium ratio. This disparity could potentially be ascribed to the fact that the participants consisted of individuals with poorly controlled hypertension, who were more inclined to receive SRS from the China Center for Disease Control and Prevention (CDC). This initiative was implemented by the national, provincial, municipal, and county levels of the CDC, advocating the utilization of a SRS during cooking as a precautionary measure [9, 31]. Additionally, the presence of hypertension is a significant determinant in the alteration of BP induced by sodium restriction. A comprehensive analysis of multiple studies revealed that hypertensive individuals experienced a substantially greater increase in SBP compared to a mixed group of hypertensive and normotensive participants within the same study on dietary salt reduction [30]. These findings strongly indicate that, when implementing dietary sodium restriction interventions, priority should be given to hypertensive adults.

The restricted cubic spline plots provided visual evidence of a consistent upward trend correlation between sodium and BP. A meta-analysis also confirmed this relationship, highlighting that the effect of sodium reduction on BP levels was more significant among individuals with higher baseline BP [3]. Our study, using various methods of urinary sodium measurement, observed similar patterns in the relationship between sodium intake and BP within the same population. However, the magnitude of this association varied depending on the specific method of urinary sodium measurement when employing the same statistical model. Previous studies have suggested that using spot urine samples to estimate sodium (Na) intake can only provide a rough average approximation of Na intake [32]. Relying on these methods to establish the connections between Na intake and BP is likely to result in biased estimations. However, despite this limitation, we proceeded with the utilization of this model as our aim was to compare the nature and extent of the relationship between sodium intake and BP, rather than solely obtaining accurate estimates. In the context of high salt intake of the Chinese population, our results support the prevention of rising BP by reducing salt intake in patients with poorly controlled hypertension.

Additional findings of the study indicate a higher prevalence of correct knowledge, attitude, and behavior among SRS users, suggesting that possessing positive and accurate beliefs and attitudes serves as a fundamental basis for modifying health-related behaviors. Notably, a considerable proportion (79.18%) of the participants demonstrated awareness regarding the detrimental consequences of excessive salt consumption. However, this knowledge did not seem to translate into effective practices for reducing salt intake, as evidenced by the frequent consumption of salty condiments by a significant portion (67.90%) of the respondents (more than 3–5 days per week). Merely relying on educational initiatives and increasing awareness is unlikely to be adequate in addressing this matter. It is preferable to actively translate this awareness into tangible actions, such as reducing the amount of salt used during cooking through the utilization of SRS or opting for salt alternatives.

The study revealed a significantly high percentage of patients (73.33%) with uncontrolled BP, similar to the findings of a survey conducted on 2198 patients in sub-Saharan countries (77.4%) [21]. Additionally, it was observed that individuals using multiple antihypertensive drugs had a significantly higher prevalence of uncontrolled hypertension (*p* < 0.001). Previous studies have indicated that the occurrence of medication errors, drug interactions, and the utilization of high-risk pharmaceuticals tend to increase with the number of medications being administered [33]. The combination of improper drug utilization and excessive salt intake has the potential to hinder hypertensive individuals in effectively managing their BP, presenting a significant opportunity for intervention in China. Our research findings demonstrate that the implementation of SRS during the cooking process is linked to a decrease in the progression of hypertension, thereby endorsing the SRS-based approach for individuals whose primary source of sodium consumption is domestic culinary practices.

There are limitations to the study. Firstly, it is important to acknowledge that this study possesses a cross-sectional design, thereby imposing inherent limitations on the capacity to establish causality. Consequently, we are unable to ascertain the causal relationship between sodium intake and BP, nor comprehend the underlying mechanism through which sodium intake may impact blood pressure. Secondly, the considerable variability in daily salt consumption among individuals introduces the potential for measurement error when converting spot urine sodium measurements to estimated 24-h urinary excretion. To avoid possible bias and enhance the accuracy of estimating sodium excretion, it is recommended to randomly collect spot or 24-h urine samples and repeatedly. The scope of our study was restricted to individuals with poorly controlled hypertension residing in Eastern China. However, it is important to acknowledge that the findings of this study may not be generalizable to populations from different regions within the country or elsewhere, as well as individuals without hypertension. However, it is important to acknowledge that in this particular population, the estimated 24-h urinary excretion may not adequately reflect actual salt intake due to the significant participant burden associated with complete collection. It is advisable for each population to develop and validate their own formula to accurately assess sodium intake from spot urine samples. Thirdly, the findings from a survey utilizing questionnaires to assess smoking habits and physical activity failed to demonstrate any statistically significant correlation with BP status. It was observed that knowledge, attitude, and behavior were somewhat linked to the utilization of SRS, although this association may be influenced by societal expectations.

This study possesses several notable strengths, primarily its extensive sample size consisting of community-based patients stratified based on their hypertension status. Previous research has demonstrated the efficacy of salt reduction in cooking for lowering BP, yet only a limited number of studies have taken into consideration the participants' hypertension status or their use of antihypertensive medication when summarizing the outcomes. Furthermore, our study was bolstered by the support of an organized multidisciplinary collaborative network, which facilitated the involvement of Chinese cardiologists and ultimately benefited patients with inadequately controlled hypertension.

## Conclusions

Hypertension remains poorly controlled in China, with its status being linked to excessive sodium consumption. The implementation of SRS strategies could potentially lead to a reduction in daily salt intake for blood pressure control among hypertensive patients. SRS users were observed to possess a moderate comprehension of the risks associated with excessive salt consumption, although their attitudes and behaviors towards salt reduction were inconsistent. To effectively decrease the prevalence of hypertension, a comprehensive campaign should encompass the promotion of knowledge, attitudes, and behaviors aimed at reducing dietary salt intake, in conjunction with the utilization of sodium reduction strategies.

## Data Availability

The datasets generated and analyzed during the current study are available from the corresponding authors upon reasonable request.

## Abbreviations

SRS: Salt-restriction spoon
WHO: World Health Organization
CVD: Cardiovascular diseases
CDC: Center for Disease Control and Prevention
BP: Blood pressure
SBP: Systolic blood pressure
DBP: Diastolic blood pressure
BMI: Body mass index
SD: Standard deviation
CI: Confidence interval
OR: Odds ratio
